# A novel prostaglandin I_2_ agonist, ONO-1301, attenuates liver inflammation and suppresses fibrosis in non-alcoholic steatohepatitis model mice

**DOI:** 10.1186/s41232-021-00191-6

**Published:** 2022-02-01

**Authors:** Satoko Motegi, Atsunori Tsuchiya, Takahiro Iwasawa, Takeki Sato, Masaru Kumagai, Kazuki Natsui, Shunsuke Nojiri, Masahiro Ogawa, Suguru Takeuchi, Yosiki Sakai, Shigeru Miyagawa, Yoshiki Sawa, Shuji Terai

**Affiliations:** 1 Division of Gastroenterology and Hepatology, Graduate School of Medical and Dental Sciences, Niigata University, 1-757, Asahimachi-dori, Chuo-ku, Niigata, 951-8510 Japan; 2 Department of Cardiovascular Surgery, Graduate School of Medicine, Osaka University, 2-2 Yamadaoka Suita, Osaka, 565-0871 Japan

**Keywords:** ONO-1301, Prostacyclin, Prostaglandin I_2_, Prostaglandin E2, Non-alcoholic steatohepatitis

## Abstract

**Background:**

ONO-1301 is a novel long-lasting prostaglandin (PG) I_2_ mimetic with inhibitory activity on thromboxane (TX) A_2_ synthase. This drug can also induce endogenous prostaglandin (PG)I2 and PGE2 levels. Furthermore, ONO-1301 acts as a cytokine inducer and can initiate tissue repair in a variety of diseases, such as pulmonary hypertension, pulmonary fibrosis, cardiac infarction, and obstructive nephropathy. In this study, our aim was to evaluate the effect of ONO-1301 on liver inflammation and fibrosis in a mouse model of non-alcoholic steatohepatitis (NASH).

**Methods:**

The therapeutic effects of ONO-1301 against liver damage, fibrosis, and occurrence of liver tumors were evaluated using melanocortin 4 receptor-deficient (*Mc4r*-KO) NASH model mice. The effects of ONO-1301 against macrophages, hepatic stellate cells, and endothelial cells were also evaluated in vitro.

**Results:**

ONO-1301 ameliorated liver damage and fibrosis progression, was effective regardless of NASH status, and suppressed the occurrence of liver tumors in *Mc4r*-KO NASH model mice. In the in vitro study, ONO-1301 suppressed LPS-induced inflammatory responses in cultured macrophages, suppressed hepatic stellate cell (HSC) activation, upregulated vascular endothelial growth factor (VEGF) expression in HSCs, and upregulated hepatocyte growth factor (HGF) and VEGF expression in endothelial cells.

**Conclusions:**

The results of our study highlight the potential of ONO-1301 to reverse the progression and prevent the occurrence of liver tumors in NASH using in vivo and in vitro models. ONO-1301 is a multidirectional drug that can play a key role in various pathways and can be further analyzed for use as a new drug candidate against NASH.

**Supplementary Information:**

The online version contains supplementary material available at 10.1186/s41232-021-00191-6.

## Background

Non-alcoholic fatty liver disease (NAFLD) and non-alcoholic steatohepatitis (NASH) are among the most important causes of chronic liver disease and cirrhosis, mainly in South America and the Middle East, followed by the rest of Asia, the USA, and Europe. It has been estimated that the incidence of NAFLD and NASH will increase in the coming decade. While the epidemiology and demographic characteristics of NAFLD vary worldwide and NAFLD and NASH are generally detected in lean patients, most of the cases are related to lifestyle and obesity [1–3]. The most important countermeasures are the correction of lifestyle, if possible, from a younger age. However, parallel development of new drugs that prevent the progression of inflammation and fibrosis is essential [4].

To evaluate the effects of cell therapy and drugs in NASH, appropriate animal models that reflect symptoms in humans, such as obesity, insulin resistance, and liver steatosis, are needed.

Melanocortin 4 receptor-deficient (*Mc4r*-KO) mice are ideal models for evaluating the effects of cell therapy and drugs against NASH [5–7]. MC4R is expressed in the hypothalamic nuclei and regulates food intake and body weight; thus, *Mc4r*-KO mice cannot control their appetite and exhibit symptoms such as obesity, insulin resistance, and liver steatosis similar to those of NASH in humans. When a high-fat diet (HFD) was started 8 weeks after birth, steatohepatitis with fibrosis and hepatocellular carcinoma were detected around 28 weeks and 1 year after birth, respectively, in the mice [8, 9]. Using this model, we had previously evaluated the anti-inflammatory and anti-fibrosis effects of mesenchymal stem cells and their exosomes, and the mechanisms of NASH-related carcinogenesis [5–7]. Thus, this mouse model is ideal for evaluating the effects of drugs against steatosis, fibrosis, and carcinogenesis.

ONO-1301 is a unique novel long-lasting prostaglandin (PG) I_2_ mimetic with inhibitory activity on thromboxane (TX) A_2_ synthase. This drug is chemically and biologically stable because of the lack of a typical prostanoid [10–18]. ONO-1301 acts primarily as a prostacyclin receptor (IP) agonist and can induce adenosine cyclic 3′,5′-monophosphate (cAMP) elevation in target cells. However, IP agonists, when administered alone, usually lose their efficacy after repeated administration. Furthermore, they increase TXA2 production, which usually increases blood pressure and thrombosis formation. However, due to the inhibitory activity of ONO-1301 on TXA_2_ synthase, tolerance development caused by repeated administration of this IP agonist is suppressed [19]. In addition, this drug can induce endogenous PGI2; PGE2; and endogenous regenerative factors such as hepatocyte growth factor (HGF), vascular endothelial growth factor (VEGF), and stromal cell derived factor (SDF)-1, which induce tissue repair processes such as angiogenesis, anti-fibrosis, anti-apoptosis, and nitric oxide (NO) generation [10, 11, 13, 14]. Based on a few reports, we suspected that ONO-1301 would have positive effects on acute liver injury. Yin et al. reported a protective role of COX-2 derived PGs in concanavalin A (ConA)-induced liver injury. In this study, COX-2^−/−^ mice developed severe damage in the liver, and these effects were canceled by the PGE1/2 analog or the PGI2 analog [20]. Mayoral et al. also reported that COX-2 dependent PGs exert a protective effect against lipopolysaccharide-induced injury in D-galactosamine-preconditioned mice (LPS/D-DalN) and ConA-induced acute liver injury model by an antiapoptotic/antinecrotic effect by accelerating early hepatocyte proliferation [21]. Indeed, Xu et al. reported that ONO-1301 decreased serum AST and ALT levels, apoptotic liver cell numbers, and expansion of necrotic areas in liver tissues. These authors focused on ONO-1301-induced HGF and showed that neutralization of endogenous HGF could reverse the therapeutic effects of ONO-1301 [10].

In this study, we evaluated the therapeutic effects and mechanisms of action of ONO-1301 against NASH in *Mc4r*-KO mice.

## Methods

### Mice

*Mc4r*-KO mice with a C57BL/6 J background, which were provided by Joel K. Elmquist (University of Texas Southern Medical Center, Dallas, TX, USA), were used to develop NASH model mice. The mice were fed a normal diet for 8 weeks (ND; CE-2; CLEA Japan, Inc. Tokyo, Japan) and then a Western diet (WD; Research Diets, Inc., New Brunswick, NJ, USA). Typically, after 20 weeks of continuous intake of WD, the liver shows inflammation and fibrosis similar to that in human NASH. To obtain macrophages and stellate cells, C57BL/6 mice purchased from Charles River (Yokohama, Japan) were used. All animals were housed in a specific pathogen-free environment and maintained under standard conditions with a 12-h day/night cycle and access to food and water ad libitum. All animal experiments were conducted in compliance with the regulations and approval of the Institutional Animal Care Committee of Niigata University.

### ONO-1301

ONO-1301 (powder, H5001) was provided by Lind Pharma Inc. (Osaka, Japan). For in vitro experiments, ONO-1301 was dissolved in dimethyl sulfoxide (DMSO, Nacalai Tesque Inc., Kyoto, Japan) to obtain a final concentration of 0.01–0.1 μM. To examine its in vivo effect, ONO-1301 powder was mixed with WD at a 0.01% weight ratio. The ONO-1301 concentration was measured using the plasma of *Mc4r*-KO mice that were administered ONO-1301 at 0.01% w/w for 20 weeks, and the mass of the liquid chromatograph was used to evaluate ONO-1301 concentration. The plasma concentration of ONO-1301 was 19.3 ± 6.1 ng/mL (means ± standard error of measurement), which was within expected range.

### Macrophage culture and assay

Bone marrow cells collected from the femurs of 10–12-week-old C57BL/6 male mice were cultured at 37 °C in the presence of 5% CO_2_ in ultra-low attachment flasks (Corning Inc., Corning, NY, USA) in Dulbecco’s modified Eagle’s medium (DMEM)/F12 (Thermo Fisher Scientific, Waltham, MA, USA) containing 20 ng/mL macrophage colony stimulating factor-1 (Peprotech Inc., Rocky Hill, NJ, USA); the medium was changed twice weekly, as described previously [22, 23]. After 7 days, the collected macrophages were harvested and seeded in 6-well Nunc™ Cell-Culture Treated Multidishes (Thermo Fisher Scientific) at a density of 3 × 10^5^ cells/well. Then, 25 ng/mL lipopolysaccharide (LPS from *Escherichia coli* O111:B4; catalog number L2630; Sigma-Aldrich, Tokyo, Japan) and 0.01 μM ONO-1301 or DMSO (control group) were added to the cultured macrophages. After 18 h, the macrophages were harvested, and the mRNA expression levels of genes encoding pro-inflammatory factors (e.g., interleukin-6 [*Il6*], tumor necrosis factor [*Tnf-a*], monocyte chemotactic protein-1 [*Mcp-1*], and inducible nitric oxide synthase [*Inos*]) and anti-inflammatory factors (e.g., interleukin-10 [*Il10*]*,* chitinase 3-like 3 [*Ym-1*], and macrophage mannose receptor [*Cd206*]) were evaluated using real-time polymerase chain reaction (PCR) (Supplementary Table 1).

### Hepatic stellate cell culture and assay

Hepatic stellate cells (HSCs) were isolated from 35 ± 5-week-old C57BL/6 female mice. Briefly, to obtain the HSCs, the livers were digested with liver perfusion medium (Thermo Fisher Scientific) and liver digestive medium (Thermo Fisher Scientific). Non-parenchymal cells from the digested cells were fractionated using 11% HistoDenz (Sigma-Aldrich, St. Louis, MO, USA) at 2500 rpm for 20 min. After isolation, mouse HSCs were cultured on collagen type I-coated 12-well plates (AGC Techno Glass Co., Ltd., Haibara, Japan) in DMEM (Thermo Fisher Scientific) supplemented with 10% fetal bovine serum (FBS; Thermo Fisher Scientific), non-essential amino acid solution (Thermo Fisher Scientific), and penicillin-streptomycin-glutamine (Thermo Fisher Scientific). After 6 h of culture, cells were washed with PBS, the medium was changed, and ONO-1301 (0.1 μM) or DMSO (control group) was added to each well. The medium was changed 24 h later. After 72 h, HSCs were harvested, and the mRNA expression levels of genes encoding activated HSC factors (α-smooth muscle actin [*Acta2*], type I collagen alpha 1 [*Col1a1*], type III collagen alpha 1 [*Col3a1*]), and quiescent HSC factors (cytoglobin [*Ctgb*] and Hedgehog interacting protein [*Hhip*]) were evaluated using real-time PCR (Supplementary Table 1).

### Endothelial cell culture and assay

Human umbilical vein vascular endothelial cells (HUVECs) obtained from Promocell (Heidelberg, Germany) were cultured according to the manufacturer’s instructions. Cells at passages 4–6 were used for all the experiments. ONO-1301 (0.1 μM) or DMSO (control group) was added to the cultured HUVECs. The medium was replaced 24 h later with fresh media containing ONO-1301 or DMSO. After 72 h, HUVECs were harvested, and the mRNA expression levels of stromal cell-derived factor-1α [*Sdf1*], hepatocyte growth factor [*Hgf*], and vascular endothelial growth factor [*Vegf*]) were evaluated using real-time PCR (Supplementary Table 1).

### Real-time PCR

Total RNA was extracted using the RNeasy kit (Qiagen, Venlo, the Netherlands) and was reverse transcribed using a QuantiTect reverse transcription kit (Qiagen) according to the manufacturer’s instructions. Gene expression analysis was performed using pre-validated QuantiTect primers (Supplementary Table 1) with the QuantiTect SYBR reagent (Qiagen). Real-time PCR was performed using the Step One Plus Real-time PCR System (Applied Biosystems, Foster City, CA, USA). Results were obtained from five to seven replicates. The gene encoding glyceraldehyde 3-phosphate dehydrogenase (*Gapdh*) was used as an internal control (Supplementary Table 1). The fold change in relative gene expression compared to the control was calculated using the ΔΔCt method.

### Serum analyses

Blood samples were obtained from the hearts of mice at 20 and 28 weeks after starting WD feeding. Mice were anesthetized, and blood was collected by cardiac puncture for biochemical analyses in the non-fasted state. Serum alanine aminotransferase (ALT), aspartate transaminase (AST), total bilirubin (Bil), albumin (ALB), total triglyceride (TG), and total cholesterol (T-cho) levels were calculated by Oriental Yeast Co., Ltd. (Tokyo, Japan).

### Immunohistochemistry

For staining of the liver tissue, 10% formalin-fixed tissue was sliced into 4-μm-thick sections. Immunohistochemistry for F4/80 (ab111101; rabbit monoclonal to F4/80, dilution 1/80; Abcam, Cambridge, UK) was performed as follows. The dewaxed tissues were subjected to antigen retrieval in 10 mM sodium citrate buffer (pH 6.0) for 20 min using a microwave. Endogenous peroxidase activity was blocked by treatment with 3% hydrogen peroxide (H_2_O_2_; FUJIFILM Wako Pure Chemical Corporation, Osaka, Japan) in PBS for 10 min at room temperature, followed by avidin-biotin blocking. The primary antibody was applied overnight in an antibody diluent reagent solution (Thermo Fisher Scientific). The secondary antibody reaction was performed using the Vecstain ABC kit (Vector Laboratories, Burlingame, CA, USA). The sections were stained by reaction with DAB TRIS tablets (Muto Pure Chemicals, Tokyo, Japan). The number of hepatic crown-like structures (hCLS) and histological features of macrophages in the liver from NASH were counted in at least 10 fields at × 200 magnification of each F4/80-stained section and expressed as the mean number/mm^2^.

### Sirius Red staining

To quantify fibrosis, liver tissues were collected at 20 and 28 weeks after the start of WD feeding. Tissues were fixed with 10% formalin, sliced into 4-μm-thick sections, and stained with Sirius Red. The images of each section were randomly (20 fields at × 200 magnification/mouse) captured using a BZ-9000 microscope (Keyence, Osaka, Japan), and quantitative analysis of the fibrotic area was performed using the ImageJ software (version 1.6.0 20, National Institutes of Health, Bethesda, MD, USA).

### Hydroxyproline assay

The levels of hydroxyproline, which is a representative collagen component, were determined in mouse livers at 20 and 28 weeks after starting WD feeding. Briefly, liver samples (20 mg) were homogenized and subjected to QuickZyme hydroxyproline assays (QuickZyme Bioscience, Zernikedreef, the Netherlands) according to the manufacturer’s protocol. Liver tissue samples were extracted, and the absorbance was measured at 570 nm. Data are expressed as the amount of hydroxyproline per mg liver tissue.

### Measurement of cAMP accumulation

cAMP was quantified from liver homogenates using a Direct cAMP ELISA Kit (ab133051; Abcam, Cambridge, UK) according to the manufacturer’s protocol and normalized to total liver weight. Intracellular cAMP concentration of cultured macrophages and HUVECs was quantified 30 min after the start of stimulation using the Direct cAMP ELISA Kit (Enzo Life Sciences, Farmingdale, NY, USA) according to the manufacturer's protocol.

### Statistical analysis

Statistical analysis was performed using GraphPad Prism9 software (GraphPad Software Inc., La Jolla, CA, USA). Data are presented as means ± standard deviation (SD). The results were assessed using Welch’s *t*-test. Differences between groups were analyzed using Welch’s one-way analysis of variance (ANOVA). Differences were considered significant at *p* < 0.05.

## Results

### ONO-1301 ameliorates liver damage and fibrosis progression in NASH model mice

**Fig. 1:**
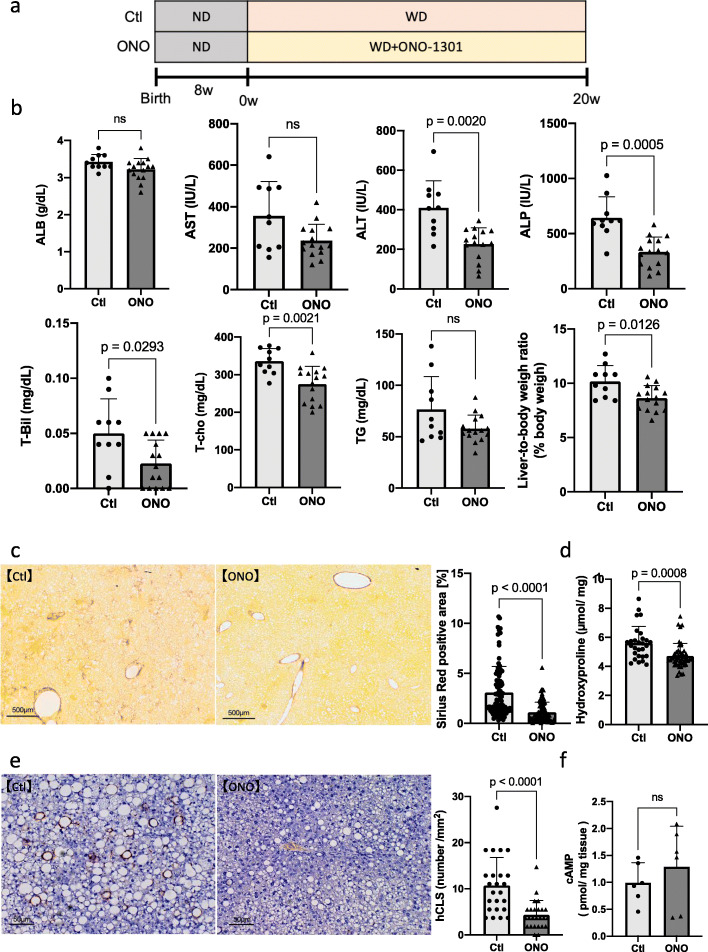
Therapeutic effects of ONO-1301 (20 weeks of treatment) in the *Mc4r*-KO NASH model mice. *Mc4r*-KO mice were fed a Western diet from 8 weeks of age and followed up for an additional 20 weeks. **A** Schematic of the experiment. **B** Serum levels of albumin (ALB), aspartate transaminase (AST), alanine transaminase (ALT), alkaline phosphatase (ALP), total bilirubin (T-Bil), total cholesterol (T-cho), and triglyceride (TG). Liver-to-body weight ratios were analyzed. **C** Sirius Red staining of liver tissues. Scale bar = 500 μm. The quantification of Sirius Red staining. **D** Quantification of hydroxyproline. **E** Immunohistochemistry of F4/80 and quantification of hepatic crown-like structures (hCLS). **F** The levels of cAMP in the liver tissues. Scale bar = 50 μm. Total number of mice in each group: *n* = 6–10 in Ctl group, *n* = 6–15 in ONO group. Data are presented as mean ± standard deviation. ns: not significant

To evaluate the therapeutic effects of ONO-1301, it was mixed into mice feed and administered to *Mc4r*-KO NASH model mice from 8 weeks after birth (ONO group). Serum biochemical levels and fibrosis accumulation were evaluated in comparison with the levels in the WD feeding control group (Ctl group) (Fig. 1a).

Analyses of serum biochemical parameters and liver-to-body weight ratio revealed that serum levels of ALT (Ctl group: 410 ± 136.8 IU/L, ONO group: 226.3 ± 82.7 IU/L, *p* = 0.0020), ALP (Ctl group: 643.4 ± 190.5 IU/L, ONO group: 333.7 ± 135.5 IU/L, *p* = 0.0005), T-cho (Ctl group: 335.7 ± 33.9 mg/dL, ONO group: 274.8 ± 47.1 mg/dL, *p* = 0.0021), and the liver-to-body weight ratio (Ctl group: 10.17 ± 1.46 g, ONO group: 8.63 ± 1.16 g, *p* = 0.0126) were significantly lower in the ONO-1301 feeding group compared to those in the control group (Fig. 1b). Evaluation of fibrosis demonstrated that the Sirius Red stained area (Ctl group: 3.10% ± 2.59%, ONO group: 1.12% ± 1.01%, *p* < 0.0001; Fig. 1c) and hydroxyproline levels (Ctl group: 5.61 ± 1.15 nmol/mg, ONO group: 4.71 ± 0.86 nmol/mg, *p* = 0.0008; Fig. 1d) were significantly decreased in the ONO-1301 feeding group compared to those in the control group. Moreover, ONO-1301 significantly reduced the number of hCLS, indicating that the macrophages aggregated around hepatocytes with large lipid droplets in the liver (Fig. 1e). In addition, we confirmed that cAMP levels in the liver tissues were tended to increase after ONO-1301 treatment (Fig. 1f). These results revealed that oral administration of ONO-1301 effectively ameliorated liver damage and fibrosis.

### ONO-1301 was effective regardless of status of NASH and suppressed liver tumor formation

**Fig. 2:**
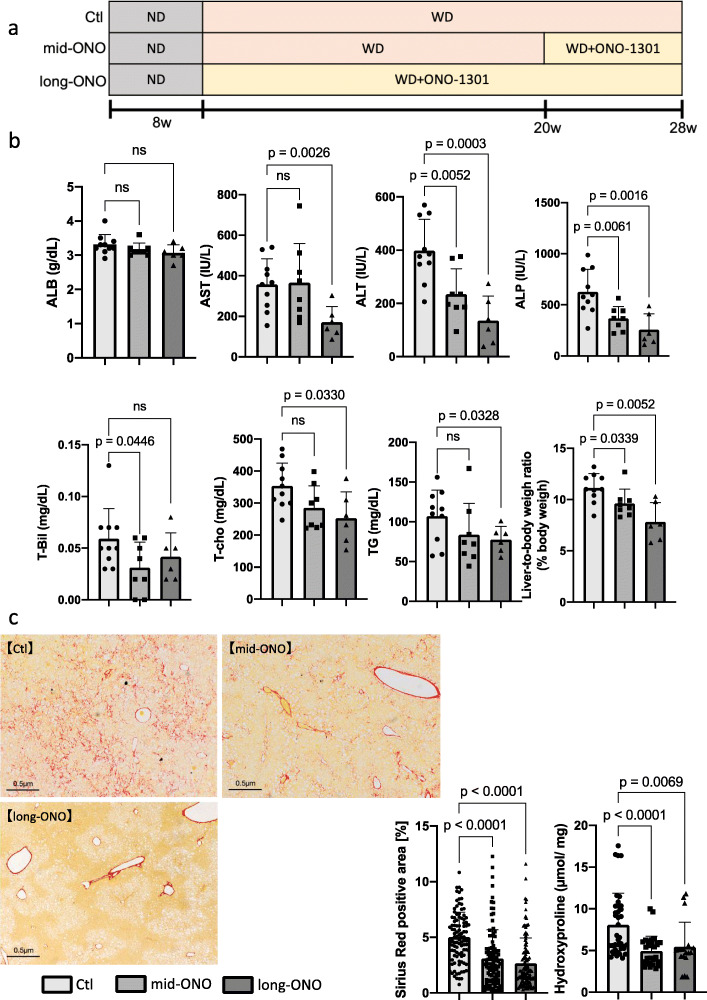
Therapeutic effects of ONO-1301 (8- or 28-week treatment) in *Mc4r*-KO NASH model mice. *Mc4r*-KO mice were fed a Western diet from 8 weeks of age and followed up for an additional 28 weeks. **A** Schematic of the experiment. **B** Serum levels of albumin (ALB), aspartate transaminase (AST), alanine transaminase (ALT), alkaline phosphatase (ALP), total bilirubin (T-Bil), total cholesterol (T-cho), and triglyceride (TG). Liver-to-body weight ratios were analyzed. **C** Sirius Red staining and **D** quantification of hydroxyproline levels. Total number of mice in each group: *n* = 10 in Ctl group, *n* = 8 in mid-ONO group, *n* = 6 in long-ONO group. Scale bar = 500 μm. Data are presented as mean ± standard deviation. ns: not significant

Next, to evaluate whether the therapeutic effect of ONO-1301 varied depending on the status of NASH, we divided the mice into three groups as follows: In the Ctl group, the *Mc4r*-KO mouse fed ND until 8 weeks, following which WD was fed until 28 weeks after birth. In the Mid-ONO group, mice fed ND until 8 weeks after birth were then fed WD for 20 weeks, followed by WD with ONO-1301 for 8 weeks under conditions similar to those of the Ctl group. In the Long-ONO group, mice fed ND until 8 weeks after birth, followed by WD with ONO-1301 for 28 weeks (Fig. 2a).

While serum levels of ALB, AST, T-cho, and TG did not change significantly in the mid-ONO group compared to those in Ctl group, serum levels of ALT (Ctl group: 397.8 ± 118.3 IU/L, mid-ONO group: 234.5 ± 95.7 IU/L, *p* = 0.0052) and ALP (Ctl group: 626.9 ± 219.6 IU/L, mid-ONO group: 367.1 ± 116.0 IU/L, *p* = 0.0061) and the liver-to-body weight ratio (Ctl group: 11.12 ± 1.41 g, mid-ONO group: 9.64 ± 1.38 g, *p* = 0.0399) in the mid-ONO group were decreased compared to those in the control group (Fig. 2b). These results revealed that ONO-1301 is effective against established NASH. Furthermore, analyses of serum biochemical parameters and liver-to-body weight ratio revealed that serum levels of AST (Ctl group: 356.7 ± 126.7 IU/L; long-ONO group: 171.2 ± 76.5 IU/L, *p* = 0.0026), ALT (Ctl group: 397.8 ± 118.3 IU/L, long-ONO group: 135.8 ± 91.5 IU/L, *p* = 0.0003), ALP (Ctl group: 626.9 ± 219.6 IU/L, long-ONO group: 256.0 ± 156.6 IU/L, *p* = 0.0016), T-cho (Ctl group: 353.8 ± 71.0 IU/L, long-ONO group: 253.2 ± 82.1 IU/L, *p* = 0.033), and TG (Ctl group: 107.0 ± 32.9 IU/L, long-ONO group: 77.5 ± 16.6 IU/L, *p* = 0.0328), and liver-to-body weight ratio (Ctl group: 11.12 ± 1.41 g, long-ONO group: 7.83 ± 1.86 g, *p* = 0.005) in the long-ONO group decreased significantly compared to those in the Ctl group (Fig. 2b). These results confirmed the sustainable effects of ONO-1301 and that the long-term use of ONO-1301 improved lipid-related markers.

**Fig. 3:**
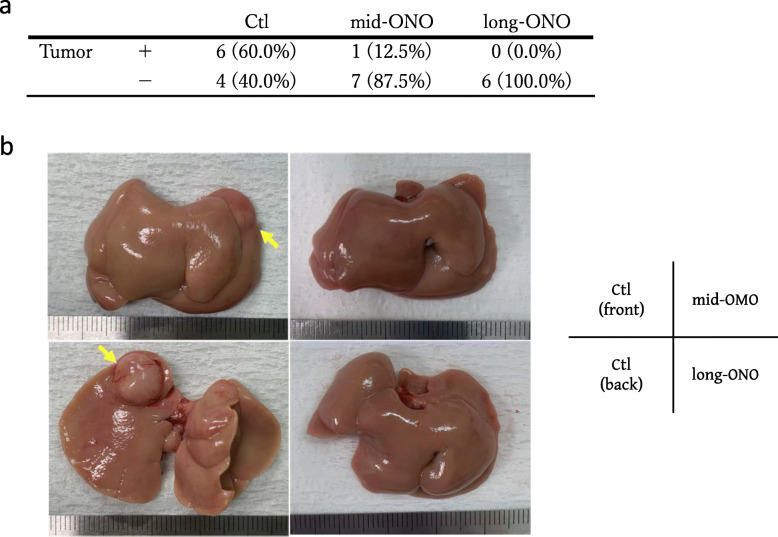
Evaluation of liver tumor occurrence in *Mc4r*-KO NASH model mice at 36 weeks of age. **A** Tumor incidence in each group. **B** Macroscopic images of the liver tumors. Total number of mice in each group: *n* = 10 in Ctl group, *n* = 8 in mid-ONO group, *n* = 6 in long-ONO group. Yellow arrows indicate the liver tumor

Evaluation of fibrosis demonstrated that the Sirius Red-stained area (Ctl group: 5.03% ± 2.21%, mid-ONO group: 3.09% ± 2.59% *p* < 0.0001, long-ONO group; 2.65% ± 2.27% *p* < 0.0001; Fig. 2c) and hydroxyproline levels (Ctl group: 8.08 ± 3.79 nmol/mg, mid-ONO group: 4.94 ± 1.79 nmol/mg, *p* = 0.0001; long-ONO group; 5.45 ± 2.94 nmol/mg *p* = 0.0069; Fig. 2d) were significantly reduced in the ONO-1301 feeding group compared to those in the control group. Finally, we evaluated the occurrence of liver tumors in the three groups and found that it was suppressed depending on the period of use of ONO-1301 (Ctl group: 6/10, 60.0 %; mid-ONO group: 1/8, 12.5 %; long-ONO group: 0/6, 0.0%) (Fig. 3a, b). These results suggested that ONO-1301 was effective regardless of NASH status and suppressed the occurrence of liver tumors.

### ONO-1301 suppressed LPS-induced inflammatory responses in cultured macrophages

**Fig. 4:**
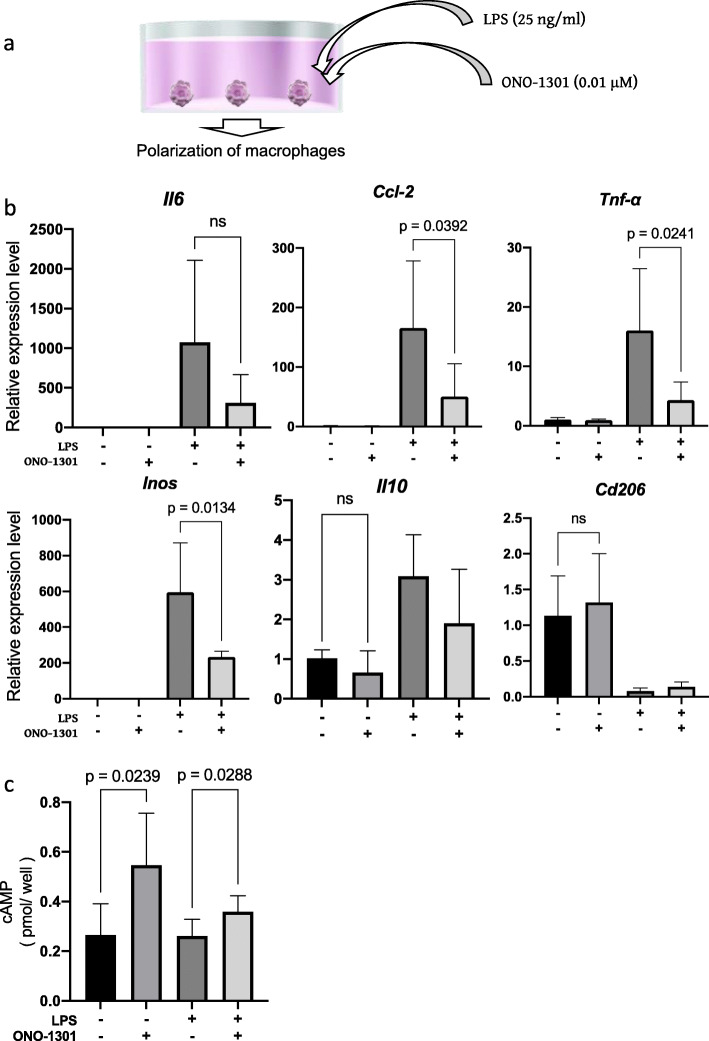
Effects of ONO-1301 against macrophages in vitro. **A** Schematic of the experiment. **B** After addition of lipopolysaccharide (25 ng/ml), ONO-1301 (0.01 μM) to macrophage cultures, mRNA expression levels of *Il6*, *Ccl2*, *Tnfa*, *Inos*, *Il10*, and *Cd206* were analyzed (each experiment was repeated seven times). **C** cAMP levels of each condition were analyzed of three independent experiments. Data are presented as mean ± standard deviation. ns: not significant

Macrophages have polarity and are key players in both pro-inflammatory and anti-inflammatory responses [24, 25]. LPS, a component of gram-negative bacteria, is known to induce pro-inflammatory effects in macrophages by upregulating the expression levels of *Il6*, *Mcp1*, *Tnfa*, and *Inos* in macrophages isolated from the bone marrow of WT mice. To confirm whether ONO-1301 can suppress pro-inflammatory effects in macrophages, ONO-1301 was added to macrophage cultures with or without LPS (Fig. 4a). LPS can upregulate the mRNA of pro-inflammatory markers *Il6, Mcp1*, *Tnfa*, and *Inos*. ONO-1301 significantly suppressed the LPS-induced pro-inflammatory markers *Mcp1*, *Tnfa*, and *Inos*. However, in the presence or absence of LPS, ONO-1301 did not affect mRNA expression levels of the anti-inflammatory macrophage markers *Il10* and *Cd206* (Fig. 4b). We also checked the cAMP levels of macrophages after adding ONO-1301 and confirmed that cAMP levels were significantly elevated after addition of ONO-1301 (Fig. 4c). These results revealed that ONO-1301 suppressed the inflammatory response of cultured macrophages as IP agonist.

### ONO-1301 suppressed HSC activation and upregulated VEGF expression

**Fig. 5:**
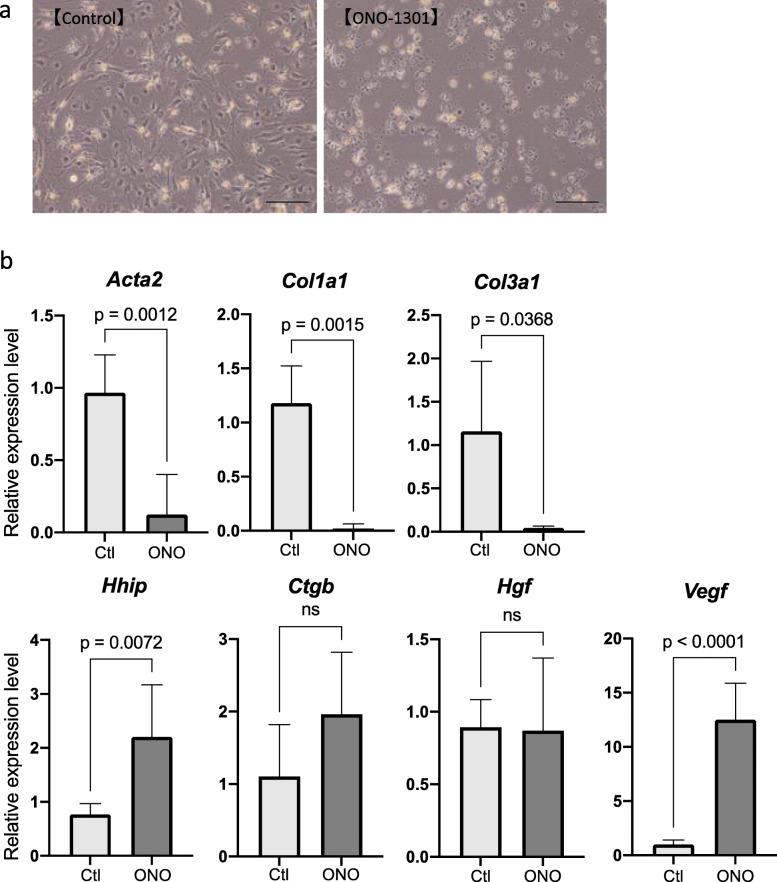
Effects of ONO-1301 against hepatic stellate cells (HSCs) in vitro. **A** Macroscopic features of HSCs on day 3 with or without ONO-1301. **B** After addition of ONO-1301 (0.1 μM) or vehicle to HSC cultures, mRNA expression levels of *Acta2*, *Col1a1*, *Col3a1*, *Hhip*, *Ctgb*, *Hgf*, and *Vegf* were analyzed (each experiment was repeated five to seven times). Scale bar = 100 μm. Data are presented as mean ± standard deviation. ns: not significant

HSCs are a major source of collagen fibers. Usually, harvested quiescent HSCs are activated when cultured on collagen-coated dishes. During this activation, HSCs produce collagen fibers and contribute to the formation of fibrosis [24, 25]. To determine whether ONO-1301 directly inhibits HSC activation, mouse HSCs were harvested and seeded on collagen-coated dishes, and after 6 h, ONO-1301 was either added (ONO group) or not (Ctl group). After 72 h of addition, the mRNA levels of *Acta2*, *Col1a1*, *Col3a1*, *Ctgb*, *Hhip*, *Vegf*, and *Hgf* were assessed using real-time PCR. Macroscopically, while spinous processes, which suggest the activation of HSCs, were detected in the Ctl group, these processes were not noticeable in the ONO group (Fig. 5a). mRNA levels of activated HSC markers *Acta2*, *Col1a1*, and *Col3a1* were significantly suppressed after the addition of ONO-1301, and the mRNA levels of the quiescent HSC marker *Hhip* were significantly higher than those in the Ctl groups (Fig. 5b). Furthermore, the expression of *Vegf* was significantly upregulated in the ONO group compared to that in the Ctl group. We could not calculate the cAMP levels due to the shortage of cells obtained from primary culture. These results revealed that ONO-1301 suppressed HSC activation and upregulated VEGF expression.

### ONO-1301 upregulates Hgf and Vegf expression in endothelial cells

**Fig. 6:**
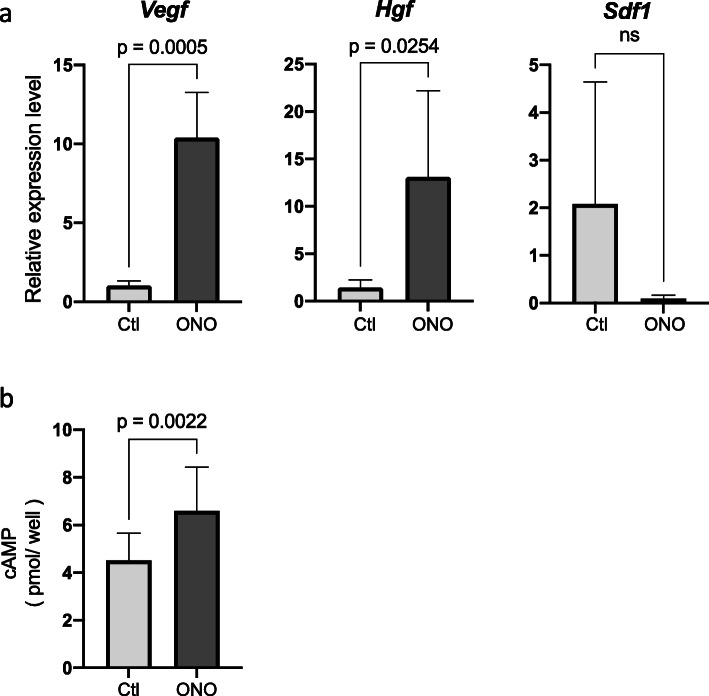
Effects of ONO-1301 against Human umbilical vein vascular endothelial cells (HUVECs) in vitro. **A** After addition of ONO-1301 (0.1 μM) or vehicle to HUVEC cultures, mRNA expression levels of *Hgf*, *Vegf*, and *Sdf1* were analyzed (each experiment was repeated six times). **B** cAMP levels of each condition were analyzed of three independent experiments. Data are presented as means ± standard deviations. ns: not significant

Finally, we examined the effects of ONO-1301 on human umbilical vein endothelial cells (HUVECs). HUVECs were cultured in 6-well plates in the absence (Ctl group) or in the presence of ONO-1301 (ONO group). After culturing the HUVECs for 72 h, *Hgf*, *Vegf,* and *Sdf1* mRNA levels were assessed using real-time PCR. ONO-1301 increased the mRNA expression of *Hgf* and *Vegf* but did not affect the mRNA expression of *Sdf1* (Fig. 6a). We also checked the cAMP levels of HUVECs after adding ONO-1301 and confirmed that cAMP levels were significantly elevated after addition ONO-1301 (Fig. 6b). These results revealed that ONO-1301 affected HUVECs as an IP agonist.

## Discussion

**Fig. 7:**
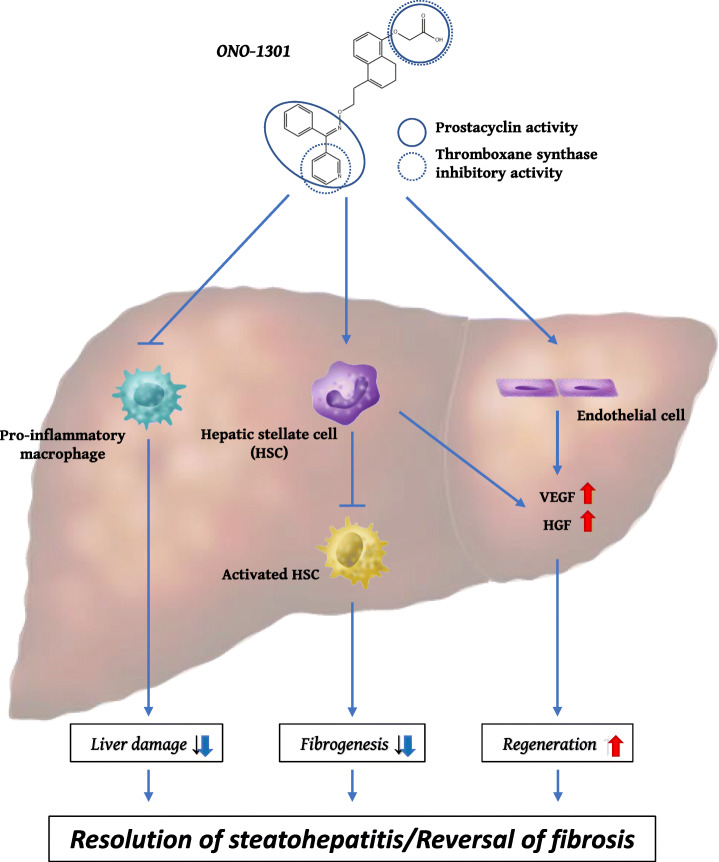
Schematic representation of the results of the current study

In this study, we demonstrated that ONO-1301 can ameliorate liver damage and fibrosis in a *Mc4r*-KO NASH mouse model. ONO-1301 had therapeutic effects regardless of the status of NASH and suppressed the occurrence of liver tumors. ONO-1301 had multidirectional effects; ONO-1301 suppressed the inflammatory responses of macrophages and activation of HSCs; it induced the production of VEGF from HSCs and that of HGF and VEGF from endothelial cells. Based on these results, we concluded that ONO-1301 induces positive effects on tissue repair in the NASH model mouse (Fig. 7).

Originally, ONO-1301 was developed as an anti-platelet drug; however, a phase I clinical study showed adverse effects such as diarrhea and headache [18]. Nevertheless, alternative medical effects of ONO-1301 as a cytokine inducer or regeneration inducer were later identified using reduced doses in vitro. Thus, ONO-1301 affects the IP receptors expressed in a variety of cells such as fibroblasts, vascular smooth muscle cells, and endothelial cells and upregulates the production of multiple factors, such as VEGF, HGF, and SDF-1, involved in tissue repair [14]. To date, the therapeutic effects of ONO-1301 have been reported in a variety of diseases, such as pulmonary hypertension, pulmonary fibrosis, arterial vascular disease, cardiac infarction [11, 18], and obstructive nephropathy [13]. Thus, this drug is expected to be used for tissue repair in a variety of diseases and organs.

There are also some reports based on which we conclude that ONO-1301 has positive effects in NASH. Some studies have reported that COX2 is upregulated in murine and human NASH livers [26–29], and Yu et al. reported that in a methionine-and choline-deficient diet (MCDD)-induced NASH model mice, the hepatic expression level of COX-2 was 10-fold higher than that in control mice [27]. Kumei et al., using IP-KO mice and a specific IP agonist and MCDD NASH model mouse, showed that PGI2-IP signaling plays a crucial role in the development and progression of steatohepatitis by modulating the inflammatory response, leading to augmented oxidative stress [30]. Furthermore, Henkel et al. reported that attenuation of PGE2 production by microsomal PGE synthase 1 ablation enhances the THF-α-triggered inflammatory response and hepatocyte apoptosis in diet-induced NASH [31]. These reports suggest that ONO-1301, a synthetic prostacyclin IP receptor agonist that can induce endogenous PGI2 and PGE2, would be effective for NASH. Indeed, in our study, using the *Mc4r*-KO NASH model mouse, ONO-1301 ameliorated liver damage and fibrosis by affecting macrophages, HSCs, and endothelial cells. Macrophages and HSCs are key players in liver inflammation and fibrosis; hence, we further investigated the effect of ONO-1301 on these cells.

Regarding the effect of macrophages, Tsai et al. reported that PGI2 analogs suppressed LPS-induced MIP-1α production in human monocytes via the IP receptor and cAMP pathway. In this study, it was confirmed that ONO-1301 had a similar effect [32]. Pan et al. reported that forced expression of prostacyclin (PGI2) synthase (PTGIS), which catalyzes the conversion of prostaglandin H2 (PGH2) to PGI2, inhibits the macrophage switch to the M1 phenotype (pro-inflammatory macrophages), and promotes M2 polarization (anti-inflammatory macrophages) [33]. Furthermore, PGE2 is known to affect macrophages by inhibiting TNF-α and other macrophage-derived chemokines [31]. Kumei et al. reported that PGI2 analog Beraprost inhibited the LPS-induced activation of macrophages and improved the pathological condition of NASH [30]. These results are consistent with our results that ONO-1301 suppressed LPS-induced inflammatory responses in cultured macrophages.

Regarding HSCs, Mallat et al. reported that an increase in cAMP induced by PGI2 and PGE2 is related to the limited proliferation of activated HSCs during chronic liver damage [34]. Pan et al. reported that PTGIS inhibits the activation of HSCs and alleviates liver fibrosis [35]. These results are consistent with our results showing that ONO-1301 suppressed HSC activation. Based on a previous study, we suspected that PGI2-IP signals are important for inhibiting HSC activation.

As shown above, the main function of ONO-1301 is that it acts as a prostacyclin receptor (IP) agonist and inhibits TXA_2_ synthase. It prevents development of tolerance caused by repeated IP agonist administration. However, in vivo, studies have reported very interesting results. Steib et al., reported that TXA2 released from activated Kupffer cells was related to portal pressure and inhibition of TXA2 reduced portal pressure in rats [36]. This indicates that ONO-1301 may contribute to prevention of liver diseases.

Recent studies have developed improvised and modified drugs from ONO-1301. ONO-1301SR, which is a poly lactic-co-glycolic acid (PLGA)-polymerized from ONO-1301, was developed to achieve a slow-releasing system of agents into target tissue, and the beneficial effects of ONO-1301SR were confirmed using various animal models of heart failure [17]. Furthermore, ONO-1301 nanospheres (ONONS) were developed to improve targeted delivery of ONO-1301 and were used to efficiently treat pulmonary artery hypertension [18]. These modified drugs decreased previously detected adverse effects in human clinical trials.

This current study has some limitations. Although the *Mc4r*-KO mouse model is an ideal animal model for NASH, it does not completely recapitulate the characteristics of NASH in humans and requires a prolonged time to mimic the conditions of human NASH. Furthermore, it is ideal to use the macrophages, HSCs, and sinusoidal endothelial cells from NASH livers. However, bone marrow-derived macrophages were used to evaluate the function of macrophages, HSCs from old wild-type female mice were used to evaluate inactivated HSCs, and HUVECs were used to evaluate the function of endothelial cells. These cells are of wild type mouse or human origin and do not completely reflect the function of macrophages, HSCs, and sinusoidal endothelial cells in NASH livers.

## Conclusions

Our study highlights the potential of ONO-1301 against liver inflammation, fibrosis, and tumor formation in NASH. ONO-1301 also significantly altered the mRNA expression of factors involved in tissue repair, further demonstrating its potential against liver damage. In conclusion, ONO-1301, which has multiple functions (anti-inflammatory, anti-fibrosis, and production of multiple factors), is an attractive drug for treating liver diseases, including NASH, which often accompanies heart, kidney, and lung diseases.

## Supplementary Information

**Additional file 1: Supplementary Table 1.** List of primers.

## Data Availability

All data needed to evaluate the conclusions in the paper are presented in the paper and/or the Supplementary Materials. Additional data related to this study are requested from the authors.

## Abbreviations

NAFLD: Non-alcoholic fatty liver disease
NASH: Non-alcoholic steatohepatitis
PG: Prostaglandin
TX: Thromboxane
IP: Prostacyclin receptor
cAMP: Adenosine cyclic 3′,5′-monophosphate
HSC: Hepatic stellate cell
VEGF: Vascular endothelial growth factor
HGF: Hepatocyte growth factor
SDF-1: Stromal cell derived factor-1
NO: Nitric oxide
WD: Western diet
DMSO: Dissolved in dimethyl sulfoxide
DMEM: Dulbecco’s modified Eagle’s medium
LPS: Lipopolysaccharide
RT-PCR: Real-time polymerase chain reaction
FBS: Fetal bovine serum
mRNA: Messenger RNA
*Il6*: Interleukin-6
*Tnfa*: Tumor necrosis factor alpha
*Mcp1*: Monocyte chemotactic protein-1
*Inos*: Inducible nitric oxide synthase
*Ym1*: Chitinase 3-like 3
*Il10*: *Interleukin-10*
*Cd206*: Macrophage mannose receptor
*Acta2*: Alpha-smooth muscle actin
*Col1a1*: Type I collagen alpha 1
*Col3a1*: Type III collagen alpha 1
*Ctgb*: Cytoglobin
*Hhip*: Hedgehog interacting protein
HUVEC: Human umbilical vein vascular endothelial cell
hCLS: Hepatic crown-like structures
ALT: Alanine aminotransferase
AST: Aspartate transaminase
Bil: Total bilirubin
ALB: Albumin
TG: Total triglyceride
T-cho: Total cholesterol
ConA: Concanavalin A
