# Supplementary material for: A novel prostaglandin I2 agonist, ONO-1301, attenuates liver inflammation and suppresses fibrosis in non-alcoholic steatohepatitis model mice
Source: Inflamm Regen. 2022 Feb 1;42:3. doi: 10.1186/s41232-021-00191-6 (PMC8805395; doi:10.1186/s41232-021-00191-6)
Supplement: Supplementary file 1 — Additional file 1: Supplementary Table 1. List of primers. [file 41232_2021_191_MOESM1_ESM.docx]

**Supplementary Table 1.** List of primers used for real-time PCR.

| Primer | Catalog number | Species | Company |
| --- | --- | --- | --- |
| *Gapdh* | QT01658692 | Mouse | Qiagen |
| *Gapdh* | QT00079247 | Human | Qiagen |
| Pro-inflammatory macrophage factors | | | |
| *Il6* | QT00098875 | Mouse | Qiagen |
| *Tnf-a* | QT00104006 | Mouse | Qiagen |
| *Inos* | QT01547980 | Mouse | Qiagen |
| *Ccl-2* | QT00167832 | Mouse | Qiagen |
| Anti-inflammatory macrophage factors | | | |
| *Il-10* | QT00106169 | Mouse | Qiagen |
| *Cd206* | QT00103012 | Mouse | Qiagen |
| Activated-HSC factors | | | |
| *Acta2* | QT00140119 | Mouse | Qiagen |
| *Col1a1* | QT00162204 | Mouse | Qiagen |
| *Col3a1* | QT01055516 | Mouse | Qiagen |
| Quiescent HSC factors | | | |
| *Hhip* | QT00147518 | Mouse | Qiagen |
| *Ctgb* | QT00124621 | Mouse | Qiagen |
| Regenerative factors | | | |
| *Hgf* | QT00158046 | Mouse | Qiagen |
| *Hgf* | QT00065695 | Human | Qiagen |
| *Vegf* | QT00160769 | Mouse | Qiagen |
| *Vegf* | QT01010184 | Human | Qiagen |
| *Sdf1* | QT00087591 | Human | Qiagen |
